# CRISPR/Cas9 in zebrafish: an efficient combination for human genetic diseases modeling

**DOI:** 10.1007/s00439-016-1739-6

**Published:** 2016-11-02

**Authors:** Jiaqi Liu, Yangzhong Zhou, Xiaolong Qi, Jia Chen, Weisheng Chen, Guixing Qiu, Zhihong Wu, Nan Wu

**Affiliations:** 1 Department of Orthopaedic Surgery, Peking Union Medical College Hospital, Peking Union Medical College and Chinese Academy of Medical Sciences, No. 1 Shuaifuyuan, Beijing, 100730 China; 2 Beijing Key Laboratory for Genetic Research of Skeletal Deformity, Beijing, China; 3 Department of Breast Surgical Oncology, National Cancer Center/Cancer Hospital, Chinese Academy of Medical Sciences and Peking Union Medical College, Beijing, China; 4 Medical Research Center of Orthopaedics, Chinese Academy of Medical Sciences, Beijing, China; 5 Tsinghua University Medical School, Beijing, China; 6 Department of General Surgery, Nanfang Hospital, Southern Medical University, Guangzhou, China; 7 Department of Central Laboratory, Peking Union Medical College Hospital, Peking Union Medical College and Chinese Academy of Medical Sciences, No. 1 Shuaifuyuan, Beijing, 100730 China

## Abstract

The next-generation sequencing identifies a growing number of candidate genes associated with human genetic diseases, which inevitably requires efficient methods to validate the causal links between genotype and phenotype. Recently, zebrafish, with sufficiently high-throughput capabilities, has become a favored option to study human pathogenesis. In addition, CRISPR/Cas9-based approaches have radically reduced the efforts to introduce targeted genome engineering in various organisms. Here, we systemically review the basic considerations in the design of gene editing in zebrafish with CRISPR/Cas9, and explore the potential of the combination of these two to support efficient functional analysis of human genetic variants.

## Introduction

Since the publication of sequenced human genome (International Human Genome Sequencing Consortium 2004), genetic variants in patients with various human genetic diseases can be rapidly identified by genome-wide analysis such as whole exome sequencing (WES), whole genome sequencing (WGS) and genome-wide association studies (GWAS), etc. (Do et al. 2012; Welter et al. 2014). Diagnostic pipelines based on WES have been established and validated in certain clinical laboratories to identify sequence variants in patients with suspected genetic disorders, but only 25% of the cases have achieved the genetic diagnosis (Yang et al. 2013). In those undiagnosed cases, etiologic mutations may be located in noncoding regions that cannot be detected by means of WES. Correspondingly, recent developments in WGS have also been increasingly applied within both the medical genetic research and the clinical practice (Knoppers et al. 2015). Additionally, gains in the diagnostic rate will be achieved through improved detection of copy-number variation which contribute substantively to disease burden (Stankiewicz and Lupski 2010; Wu et al. 2015). Even so, analysis of NGS data alone is normally insufficient to distinguish disease-causing sequence variants from the many potentially functional variants, and false assignments of variant pathogenicity would seriously impede our biological understanding of disease (Richards et al. 2015). Recent study has shown that even rare homozygous loss-of-function (rhLOF) variants could be extensively revealed by NGS in healthy individuals and may not always be as clinically relevant as often considered (Narasimhan et al. 2016). In this case, functional studies showing analogous phenotypes in well-established cell or animal models by editing the homologous genes are strongly required to validate the pathogenic causality of the specific genes or variants (MacArthur et al. 2014).

Over the past three decades, the mouse (*Mus musculus*) has always been considered to be the necessary preclinical model to study disease states and test new therapies (Dow and Lowe 2012). However, its utility in performing high-throughput analysis is challenging considering their small number of progeny and relatively high cost. By contrast, a small aquatic vertebrate, the zebrafish (*Danio rerio*), is rapidly becoming a new popular option in translational research (Gama Sosa et al. 2012). Sequencing of zebrafish has just been completed by the UK Sanger Institute and revealed that approximately 70% of human genes had functional homologs in zebrafish, suggesting most human pathogenesis could be modeled in zebrafish (Howe et al. 2013). And several logistical advantages of zebrafish have been gradually recognized and propelled its rise as an attractive model, including high fecundity, cheap husbandry, external fertilization, rapid development, transparency of embryos and larvae, as well as ease of experimental manipulations. Therefore, the zebrafish may represent an ideal model for medium and high-throughput genetic research (Lieschke and Currie 2007).

Recent years have seen the continuous development of several types of tools for DNA manipulation, including zinc finger nucleases (ZFNs), transcription activator-like effector nucleases (TALENs), and the clustered regularly interspaced short palindromic repeat (CRISPR) systems (Gaj et al. 2013). They immensely facilitate the wide application of genome editing in various organisms, featured by high site specificity, flexible design, and ease of operation. Particularly, CRISPR/CRISPR-associated protein 9 (Cas9) systems is the most rapidly developing class, which can be easily targeted to virtually any genomic location of interest by a customizable short RNA guide (Mali et al. 2013). In fact, the utility of CRISPR/Cas9 system extends far beyond functional genome editing such as knock-out and knock-in of individual genes. Combined with specific functional effector domains, desired perturbations could be allowed, such as transcriptional control, epigenetic modulation, DNA labeling or inducible regulation (Hsu et al. 2014). And here, we mainly review the basic considerations for editing coding genes to uncover their function, especially for those revealed by NGS study of human pathogenesis. Recent successes and existing challenges in this field is summarized, and we particularly emphasize the developing utility of CRISPR/Cas9 system in the zebrafish platform for the study of human genetic diseases.

## Zebrafish: a prepared model for studying human pathogenesis

As early as the 1930s, the zebrafish emerged as a classical developmental and embryological model in biomedical research. Since then, numerous important observations have been first made to answer the questions of vertebrate development by taking advantage of its embryological manipulability (Grunwald and Streisinger 1992; Amsterdam et al. 1999). In the 1990s, thousands of fish mutants related to early embryonic development were identified through the two large-scale random mutagenesis screenings without sophisticated infrastructure, and utilizing these attributes, the zebrafish was established as a mainstream model in development biology (Driever et al. 1996; Haffter et al. 1996). Early forward genetic screens carried out in zebrafish relied on the use of chemical DNA mutagens (ENU), followed by the isolation and characterization of fish individuals with the phenotypes of interest (Patton and Zon 2001). These experiments established the zebrafish as a classical model to investigate the genetics of embryonic patterning and development, since the phenotypes of these gene perturbations were easily noticeable and characterized (Amsterdam and Hopkins 2006). However, three major disadvantages have been realized that limited the use of these random mutagenesis schemes: first, the positional cloning of the causal mutations can be costly and laborious. Second, random mutagenesis usually generates heterozygous mutants, and recessive inherited phenotypes may fail to be detected in these screenings. Finally, it is impossible to inactivate every gene in the genome with the random mutagenesis, which means the depth and integrity of the genetic screens are inherently limited.

Soon after that, retroviral integrations were applied in zebrafish to facilitate insertional mutagenesis and transgenesis. Similar to the ENU-based random mutagenesis, large-scale genetics screens for developmental defects were conducted with retroviruses (Amsterdam et al. 2004; Varshney et al. 2013).And all the mutated genes are allowed to be identified systematically after retroviral integrations. Similarly, transposons-based gene trap or enhancer trap were also effective in the zebrafish genome, such as Tol2, Sleeping Beauty and Ac/Ds (Kawakami et al. 1998; Davidson et al. 2003; Choo et al. 2006). Due to their simplicity, high insertion efficiencies and large cargo size, they have been widely used in zebrafish in recent years.

To study the phenotypic consequences after perturbing selected genes, multiple targeted genetic approaches were developed. Fish geneticists injected the early embryos with either mRNA or antisense morpholino oligomers (MO) to generate a transient gene over-expression or knock-down, and prepared zebrafish as an accessible model for rapid confirmation of gene functions in vertebrate (Hammerschmidt et al. 1999; Nasevicius and Ekker 2000). Usually 25 bases in length, MOs are synthetic nucleic acid analogs and sterically block access of other molecules to complementary sequences of RNA by standard nucleic acid base-pairing (Summerton 1999). Injection of a MO is capable of preventing the translation of both zygotic and maternal transcripts. Importantly, recent study has shown that approximately 80% of morphant phenotypes in morpholino treated fish were not successfully recapitulated in actual genetic mutants (Kok et al. 2015). The disparity was traditionally explained by its short acting periods (typically 2–4 days) because of degradation and dilution, and the uncertain off-target effect induced by increased dose of MO, such as non-specific p53 activation (Robu et al. 2007). Importantly, functional study of specific genes seems more complicated than expected, thus encouraging us not to rely completely on MO analysis.

**Fig. 1:**
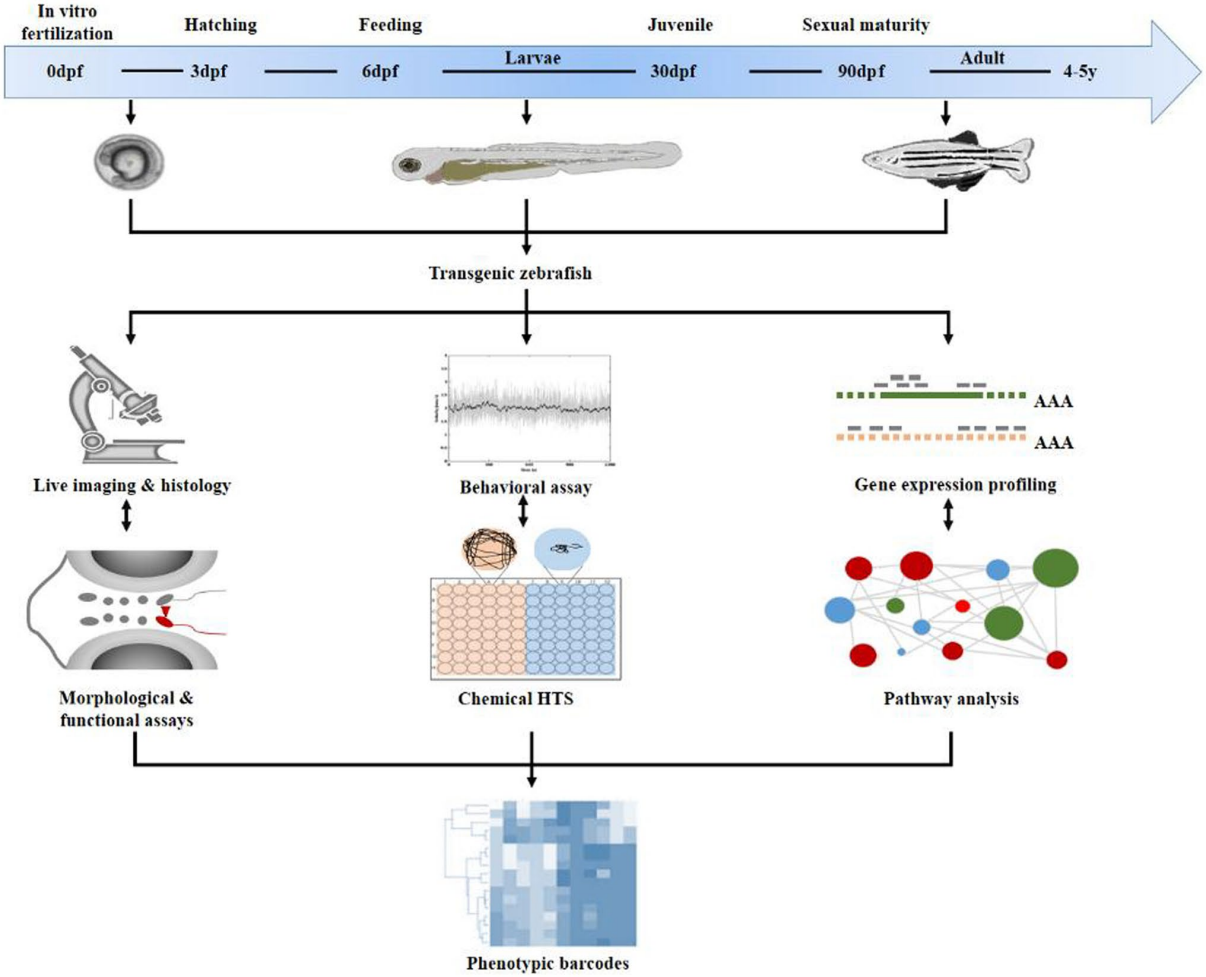
The integrated phenotyping toolbox to examine genotype-phenotype correlations in transgenic zebrafish models. The zebrafish develops and reaches sex maturity rapidly, thus time spent in gene manipulation and following phenotyping could be reduced. Once the transgenic fish is available, it is critical to apply the appropriate phenotyping tools at a right time window, which is dependent on the pathogenic features of the disease. And here we highlight the characteristic benefits of fish for imaging, behavioral assays and gene expression profiling. Quantification of the phenotypes is able to generate the phenotypic barcodes, thus assisting in high-throughput analysis or chemical screening

The advantages of zebrafish, as a genetically manipulable vertebrate model system, are reflected by their large brood size, short life cycle and easy husbandry. Hundreds of eggs could be fertilized externally every week, which are subjected to direct observations and manipulations under a microscope. Within the first five days, the optically clear fish embryos develop rapidly with no artificial feeding required, and this is the most accessible time window to study the effects of certain genetic perturbation without being confounded by environmental factors (Lieschke and Currie 2007). Zebrafish reach sexual maturity by three months of age, thus edited genes could be passed by generations from the founder lines rapidly. To examine genotype-phenotype correlations implicated in human diseases, an integrated phenotyping toolbox has been continuously under development, and here we only highlight the characteristic benefits of fish for imaging, behavioral assays and gene expression profiling (Fig. 1).

These methodological advantages, offered by fish model as inaccessible luxuries for studies on other mammalian models such as mice or rats, are critical for effective functional studies and drug discovery. The larval zebrafish is small and transparent, which allows the satisfying optical access to perform deep in vivo imaging (Fig. 1). For example, the unprecedented visualization of neuronal activity in hundreds of neurons at the same time was enabled by the whole brain functional imaging techniques developed recently (Prevedel et al. 2014). More detailed structural analysis can be conducted by whole mount histochemistry with a range of well-characterized histochemical markers. Moreover, optogenetic tools could reversibly modulate gene expressions or protein activities at the cellular or circuit level with high temporal and spatial resolution (Cosentino et al. 2015). Behavioral phenotypes are the most complex manifestations of multiple diseases, especially for those affecting the CNS or musculoskeletal systems (Stewart et al. 2014). Automated behavioral video tracking systems have been developed to quantify the fish behavior with a range of parameters on a large scale (Zhou et al. 2014). Corresponding functional behavioral tests and experimental set-ups are gradually standardized for both larval and adult zebrafish, proving the potential of zebrafish in cost-effective high-throughput screens (HTSs) and therapy development (Rihel et al. 2010) (Fig. 1). Apart from the manipulation at the genomic level, studies involving gene expression profiling are also feasible in zebrafish. This is particularly important to give mechanistic insights into the downstream events induced by the genomic perturbations. In study involving early embryonic (egg to early gastrulation) stages, maternal transcript has been shown to play important roles, which can only be investigated by genome-wide transcriptome analysis (Aanes et al. 2011). Moreover, biological differences induced by gene editing are often more than expected. In these cases, comparing genomes with proteomes and transcriptomes is critical to understand the phenotypic change in a network-based view (Rossi et al. 2015) (Fig. 1).

## CRISPR/Cas9: from adaptive immunity to genome engineering

The CRISPR system was first discovered as one of the many different antiviral defense mechanisms in prokaryotes (archaea and bacteria) against invading phages and other mobile genetic elements (Deveau et al. 2010; Horvath and Barrangou 2010; Marraffini 2015; Wright et al. 2016). The story started with the earliest detection of repeated copies downstream of the *iap* enzyme in *E. coli*, consisting of 29 nt sequences intervened by several 32 nt spacers (Ishino et al. 1987). Since then, similar arrays were sequenced and reported in other bacteria and archaea, and these interspaced sequences were named clustered regularly interspaced short palindromic repeat (CRISPR) (Mojica et al. 2000; Jansen et al. 2002). Detailed bioinformatic analysis of the spacers showed striking similarity to the sequences of certain viruses or phages that infected the particular prokaryotes without these spacers (Mojica et al. 2005). These evidences suggested that CRISPR loci might protect those prokaryotes against specific infections, as an adaptive immune system with sequence-based target specificity.

**Fig. 2:**
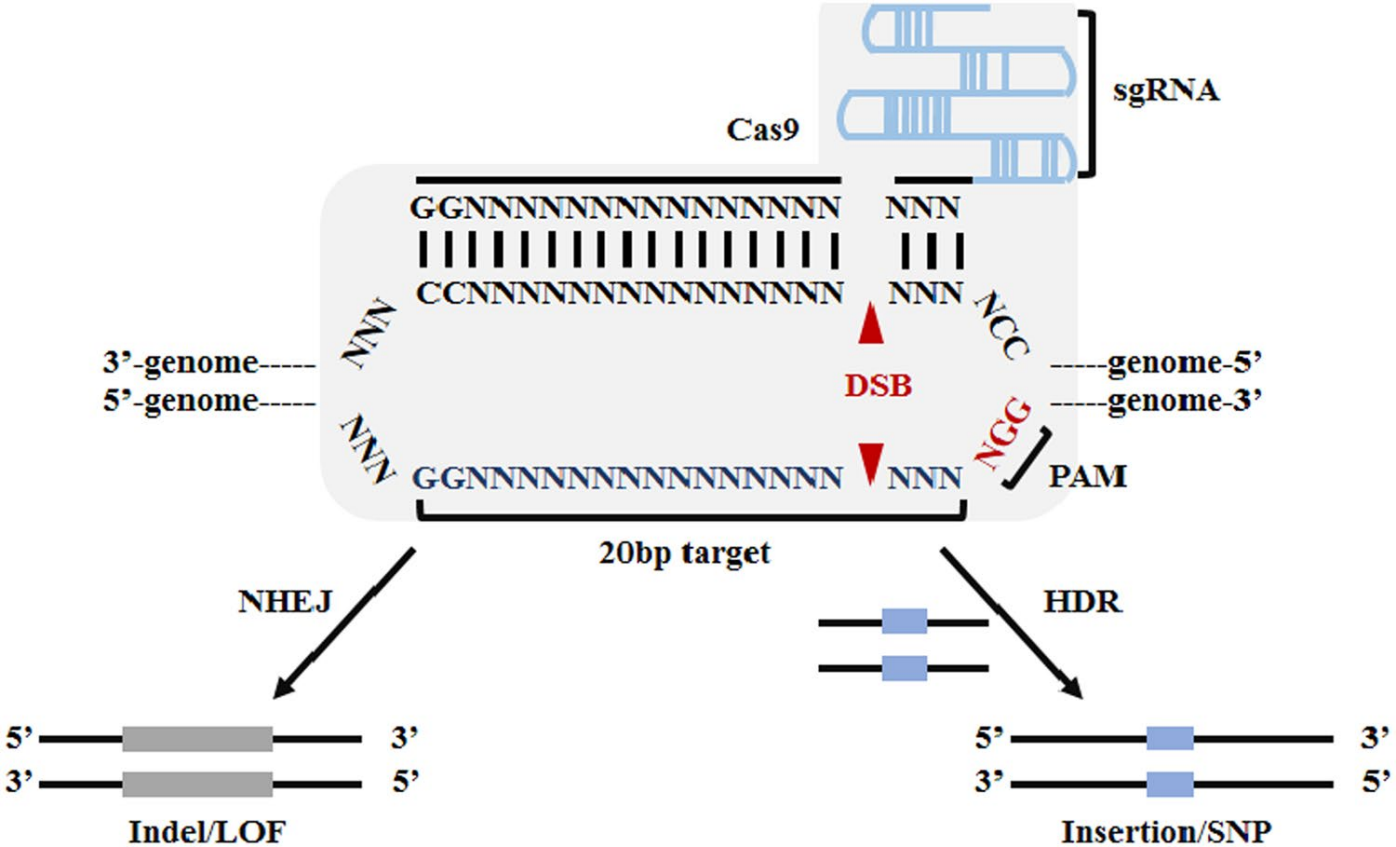
Schematic illustration of the components of engineered CRISPR-Cas9 systems. The chimeric single guide RNA (sgRNA) interacts with the complementary strand of the DNA target site harboring an adjacent protospacer adjacent motif (PAM) sequence (blue and red text, respectively), which is recognized and cleaved by Cas9 nuclease (*light gray shape*). The PAM is required for sequence specificity of Cas9-mediated endonuclease activity against genomic DNA

At the same time, analysis of the CRISPR loci revealed a conserved module adjacent to the spacers and repeats, named CRISPR-associated (cas) gene, based on which the CRISPR system was then classified into three types (I–III) (Jansen et al. 2002; Haft et al. 2005). As the studies of these three components proceeded, experimental evidences were collected to delineate the detailed mechanisms of CRISPR as sequence-based immune system (Barrangou et al. 2007). For example, in the well-characterized type II CRISPR system, spacers are derived from phage genomic sequences after viral challenge, and determine the target specificity of phage resistance by maturing into crRNA (CRISPR relative RNA), while the Cas9 nuclease provides phage resistance by cleaving virus at spacer-matching regions (Brouns et al. 2008). Three years later, the last key component in CRISPR activation, a non-coding trans-activating crRNA (tracrRNA), was uncovered to direct crRNA maturation and then facilitate RNA-guided targeting of Cas9 by base-pairing to mature crRNA (Deltcheva et al. 2011). Finally, as the CRISPR research was accelerated, the two-RNA structure formed by the hybridization of mature crRNA and tracrRNA was replaced by an engineered single guide RNA (sgRNA) to facilitate Cas9 to accomplish sequence-specific dsDNA cleavage (Jinek et al. 2012). With the combination of Cas9 and sgRNA, the integrated CRISPR system holds great promise to be engineered as a programmable and transferrable tool to accomplish genome editing (Fig. 2).

Traditional application of the CRISPR-based technology mainly refers to functional knock-out of individual genes. To accomplish this, a Cas9 protein and a sgRNA must be introduced together into each target cell by transfection or transduction. These two molecules would form a complex with the targeting DNA sites, a 20 nt sequence neighboring a protospacer adjacent motif (PAM) (Fig. 2). At this time, two endonuclease domains in the Cas9 protein produce double-stranded breaks (DSBs) in the targeted genomic sites, which are subsequently repaired through non-homologous end-joining (NHEJ). By erroneous repair of the DSBs, NHEJ tends to produce insertions or deletions (indels) mutations, therefore creating frame-shifts and loss-of-function (LOF) mutations. On the other hand, sequence templates could be introduced in the process of homology-directed repair (HDR), thus sequences of interest are inserted into the defined genomic sites (Fig. 2) (Auer et al. 2014; Li et al. 2015).

Understanding of the CRISPR-Cas functionality has achieved tremendous progress over the past few years (Mohanraju et al. 2016). Correspondingly, continuous development of engineered CRISPR-Cas variants is providing increasing scenarios for their application in genome editing (Gurumurthy et al. 2016). In addition to inducing error-prone repair of targeted DSBs, catalytically inactive Cas9 (dCas9) proteins guided by sgRNAs have been used to repress or activate gene transcription without introducing irreversible mutations to the genome, which are commonly referred to as CRISPRi/a systems (Qi et al. 2013; Gilbert et al. 2013). Recently, modified Cas9 has been proved to be able to induce programmable editing of a target base in genomic DNA without double-stranded DNA cleavage (Komor et al. 2016). And CRISPR/Cas9 also enables precise and efficient genome editing for chromosomal structural variations (SVs) research (Park et al. 2016). This is of vital importance considering both SNPs and SVs contribute to serious genetic burdens (Carvalho and Lupski 2016). The CRISPR system has been employed in genome editing first in eukaryotic cells, which was then extended to multiple animal models such as zebrafish, mice, monkey, etc (Hwang et al. 2013; Li et al. 2013; Niu et al. 2014; Benakanakere et al. 2016). Noticeably, the rapid adoption and extensive utility of the CRISPR/Cas9 technology were greatly assisted by a combination of tools and resources currently available (Graham and Root 2015).

## CRISPR/CAS9-edited zebrafish: a high-throughput approach to translational research

**Fig. 3:**
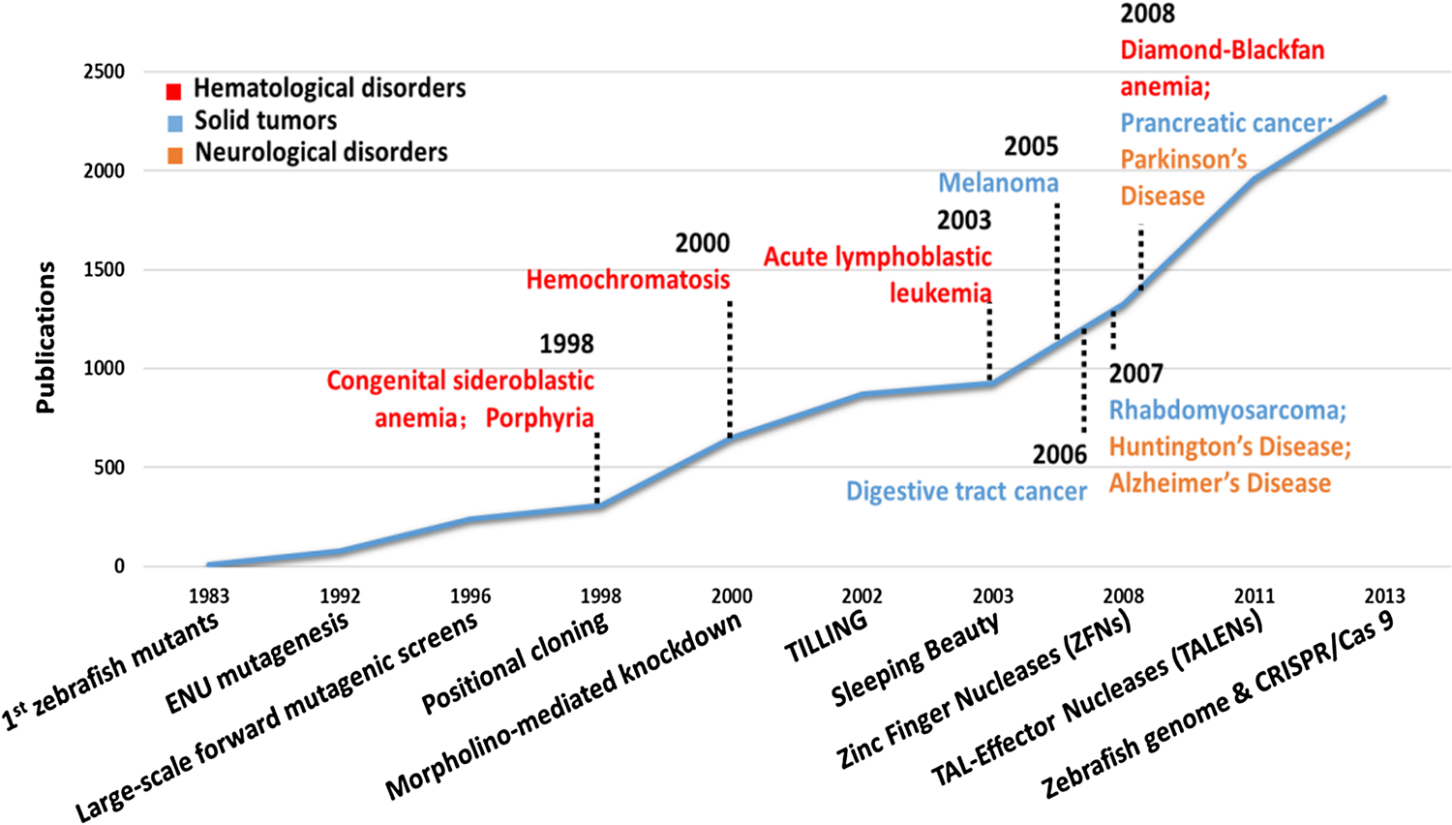
The developing utility of zebrafish in research of disease modeling. It shows above the timeline of important disease model studies and technological developments in zebrafish research. The line represents the evolution of the number of PubMed publications on zebrafish per year between 1983 and 2013. Earliest models of porphyria and other hematologic disorders could date back to 1983. As shown in the figure, the use of zebrafish in modeling human diseases has increased dramatically over the past years, benefiting from the development of several tools including morpholino, ZFNs, TALENs and CRISPR/Cas9

In the recent ten years, ZFNs, TALENs and CRISPR/Cas9 were applied in fish mutagenesis as the most successful demonstration of targeted gene inactivation (Doyon et al. 2008; Huang et al. 2011; Jao et al. 2013). These nucleases-based genome editing tools introduce gene knock-out by targeting the specific sites of interest with different recognition modules, induce precise DSBs at specific endogenous genomic loci, and finish with the error-prone DNA repair through NHEJ. The use of these gene-editing tools has proven to be revolutionary in zebrafish research, essentially making it an alternative model to address important questions in genetics, developmental biology, drug discovery, toxicology. And disease models for various genetic disorders and pathological processes have been rapidly and successfully constructed in zebrafish, including hematological disorders, malignancy, and neurological syndromes (Fig. 3) (Lieschke and Currie 2007; Ablain and Zon 2013; Kalueff et al. 2014).

Compared to ZFNs and TALENs, the easy programmability of the DNA binding domains (sgRNAs) is the most advantageous feature of CRISPR/Cas system, making it the most amenable approach to high-throughput mutagenesis projects (Table 1). Moreover, there are an increasing number of tools designed for CRISPR/Cas9 system in zebrafish. Most of them are websites or softwares designed to assemble sgRNAs with minimized off-target effects based on the wild type genomic sequences, including CRISPR MultiTargeter, CRISPRdirect, CCTop, CHOPCHOP, sgRNAcas9, CRISPRscan and so on (Xie et al. 2014; Montague et al. 2014; Prykhozhij et al. 2015; Stemmer et al. 2015; Naito et al. 2015; Moreno-Mateos et al. 2015). In addition, the newly developed HiTSelect is a comprehensive analysis pipeline for rigorously selecting screen hits and identifying functionally relevant genes and pathways by addressing off-target effects (Diaz et al. 2015). Overall, these tools notably benefit the usability of CRISPR/Cas9 editing system in zebrafish.

**Table 1:** Comparison of ZFN, TALEN and CRISPR/Cas9 techniques

Tool name	Target sequence	Recognition module	Transmission efficiency	Throughput
ZFN	Every 140–400 bp	Zinc finger domain	Low	Low
TALEN	Every 1–3 bp	TALE	Variable	Moderate
CRISPR/Cas9	N20-PAM sequence	sgRNA	High	High, proper for reverse genetic screening

*ZFN* zinc finger nuclease, *TALEN* transcription activator-like effector nuclease, *sgRNA* single guide RNA

CRISPR/Cas9 mediated gene knock-out was first performed in zebrafish by Hwang et al. with somatic mutagenesis rates ranging from 24 to 59% in 10 loci (Hwang et al. 2013). Like other reagents, characterization of its off-target effects is challenging. Previous work indicated that DSB could be induced by Cas9 even when small insertions (DNA bulge) or deletions (RNA bulge) existed in the DNA sequence compared to the RNA guide strand (Lin et al. 2014). However, whole genome sequencing to identify the off-target events of CRISPR/Cas9 in cells revealed low incidence of off-target mutations (Veres et al. 2014). Meanwhile, collective evidence showed that the off-target effect was related to the characteristics of the mismatch nucleotides, such as its number and identity (Fu et al. 2013; Hsu et al. 2013; Pattanayak et al. 2013). And 3′ end of the sgRNA sequence may be of particular importance with regard to its specificity (Cong et al. 2013). Computational tools are also developed to predict the off-target and on-target scores (Haeussler et al. 2016). However, the unbiased measurement of the off-target effects has not been performed in zebrafish. Limited insights can be gained from the recently conducted high-throughput gene targeting study using CRISPR/Cas9, in which mutations was generated in 99% of the genes tested and germline transmission was achieved in 28% of them (Varshney et al. 2015). At certain sites, the efficiency of the CRISPR/Cas9 system can reach up to 98%, and the rates of mutagenesis at potential off-target sites are low (1–3%) (Hruscha et al. 2013). However, it is still highly recommended to observe the phenotypes of the mutant fish across generations to dilute irrelevant alleles with off-target events.

**Fig. 4:**
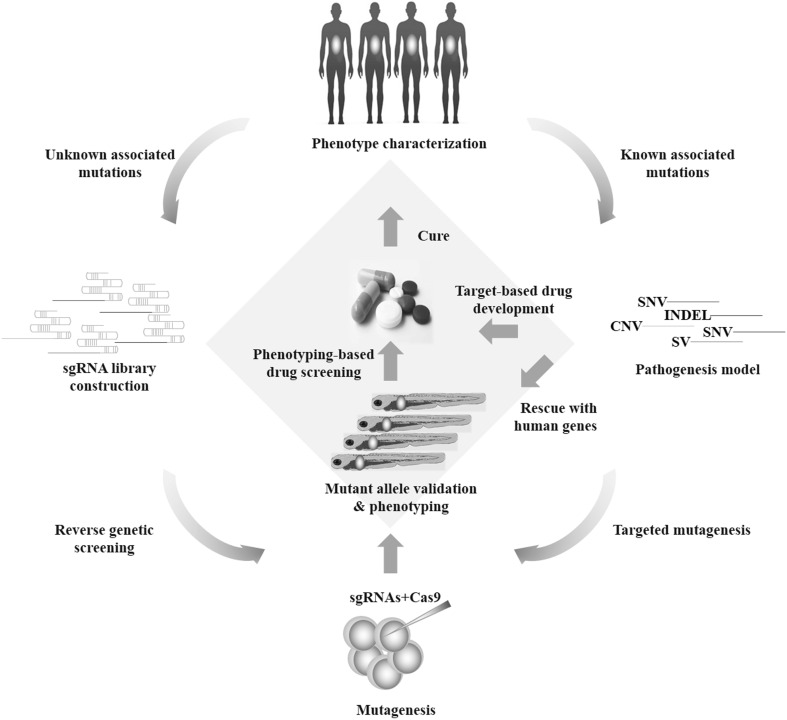
A high-throughput targeted mutagenesis pipeline to study human genetic disease with the combination of CRISPR-Cas9 system and zebrafish. Phenotypes of a certain disease or syndrome are characterized and categorized in details, as a clinical reference for animal model characterizations. Depending on the genetic research background of the disease, individual sgRNA could be constructed and injected into either the yolk or the cell of one-cell stage embryos. When information of candidate genes or mutations is limited, sgRNA library is also easily constructed in the 96-well format with one target-specific sequence and the other a generic oligonucleotide that contains the remaining nucleotides required in a sgRNA. Similarly, individual sgRNA is injected into the fish embryos. The founder fish are often outcrossed to wild type to generate heterozygous F1 (3 months) and F2 fish (6 months), and phenotype-genotype analysis is carried out in the F2 fish as shown in Fig. 1. In certain screening design, phenotyping can be performed in F0 or F1 fish to reduce the timeline, although off-target effects are more concerned in this approach. With the proper phenotypes characterized, the potential causal link between genotypes and phenotypes can be drawn. Further design of studies may include chemical screenings based on the particular phenotype in fish, thus improving our understandings of the pathogenesis and accelerating therapy development

However, a broader range of DNA sequence modifications is highly desirable such as locus-specific SNP introduction or gene insertion, considering that proposed pathogenesis models of the associated genes revealed by NGS technology can be highly diversified (Stankiewicz and Lupski 2010; Do et al. 2012; Varshney and Burgess 2014). HDR-mediated genome editing has been successfully employed in zebrafish after co-injection of a donor plasmid. However, this remains a low-efficiency process especially when integrating a relatively long DNA fragment in targeted sites (Auer et al. 2014). In this context, genome-specific knock-in techniques in zebrafish are still under improvement and intron-based knock-in approaches in zebrafish was newly developed using the CRISPR/Cas9 system (Li et al. 2015). Inspiringly, if the efficacy of CRISPR/Cas9 system is constantly improved to a reliable level, phenotypes can directly be assayed in the injected embryos. And this seems more practical since Varshney et al. designed a high-throughput targeted mutagenesis pipeline in zebrafish with the CRISPR/Cas9 system (Varshney et al. 2015). They targeted two different loci for each gene and had a 99% success rate for generating mutations across 83 genes, with half of their injected embryos transmitting mutations through the germline to the next generation. Moreover, organ-specific phenotypic screenings or functional observations could routinely be conducted in the transgenic fish with fluorescent organs, thus dramatically reducing the evidence collection time for the perturbed genes (Fig. 4) (Shah et al. 2015; Varshney et al. 2015).

In modeling diseases with zebrafish, choice between MO and CRISPR/Cas9 system is highly dependent on the specific situation. Detailed comparison of these two tools is listed in Table 2. While MO is a traditional tool that has been used in functional knock-down of numerous genes, CRIPSR/zCas9 system is an integrated toolbox to accomplish functional gene perturbations including both loss of function and gain of function experiments, with rapid development expected in the near future (Auer et al. 2014; Varshney et al. 2015). MO is designed for the exploration of phenotypes in early developmental stage of the fish. The typical time point to observe phenotypes in a CRISPR-Cas9 mediated transgenic fish is after the generation of F2, which generally takes more than six months. Recent study has showed optimal results proving the possibilities to examine phenotypes in a high-throughput manner in F1, which can be available within three months (Varshney et al. 2015). Interestingly, enormous disparity in the phenotypes between the previously reported MO treated fish and recently generated transgenic fish was reported (Kok et al. 2015). The use of MO is seemingly discouraged considering both the unrepeatable phenotypes and the easy access to the CRISPR-Cas9 systems. However, another study showed that complete gene knock-out could induce genetic compensation by other related genes, which ablated the phenotypes seen with MO (Rossi et al. 2015). And more complete gene knock-out is accomplished in MO experiments since the compensation does not happen with MO. Meanwhile, several other potential issues cannot be ignored in generating fish mutant. First, successful functional knock-out has to be validated, since alternative starting sites of transcription or splicing sites may exist in certain genes. Hypomorphic alleles may also be generated with minimal functions reserved. To be concluding, the choice between MO and CRISPR/Cas9 system is not mutually exclusive, but complementary to each other.

**Table 2:** Comparison of CRISPR/Cas9 system and MO in disease modeling with zebrafish

	CRISPR/Cas9 system	MO
Targeted loss of function	Cas9 induces indels and frame-shifts (KO); CRISPRi represses gene transcription	Translation of target genes are blocked (KD)
Targeted gain of function	Sequences are inserted with templates co-injected with Cas9 (KI); CRISPRa activates gene transcription	Unavailable
Timeframe	In KO and KI studies, phenotypes can be observed in F0 (0–5d), F1 (3 m) or F2 (>6 m), off-target effects need to be considered in early generations	Phenotypes can be observed in F0 (0–5d)
Cost and throughput	Relatively cheap and high-throughput, depending on the study design and individual institutes	Cheap and high-throughput
On-target efficacy	Highly variable depending on the design and target sequence	
Gene dosage modulation	Complete KO is accomplished with a coding frameshift. Gene dosage modulation can be done with CRISPRi/a	Complete KO is generally not available
Conditional function	Conditional gene editing is accomplished with conditional Cas9 expression	Generally not available
Duration of the effect	KO or KI is permanent and can be transmitted through generations	Transient KD
Reversibility	Cas9 KO is irreversible, CRISPRi/a is reversible	Reversible
Toxicity	Highly variable among the different sgRNAs, not correlate with the on-target efficacy	Increase with the MO dose injected

*KO* knock-out, *KD* knock-down, *KI* knock-in, *MO* morpholino oligomers

A number of successful studies have exploited the CRISPR/Cas9-mediated zebrafish to test the causal role of specific genetic perturbations in a ‘genotype-to-phenotype’ approach (Hwang et al. 2013; Jao et al. 2013; Shah et al. 2015; Varshney et al. 2015). For example, Perles et al. employed the CRISPR/Cas9-mediated zebrafish to investigate the effect of *MMP21* knock-out (Perles et al. 2015). *MMP21* gene was suggested to be associated with human heterotaxy. However, few studies had verified the underlying mechanism and its role in pathogenic pathway. Cardiac looping defects were observed in zebrafish embryos with *MMP21* deleted, together with concomitant defects in Notch signaling. Moreover, the precision of CRISPR/Cas9 editing is utilized to isolate phenotype-causing genes in difficult genomic regions. Cloche is a gene that plays critical roles in haemato-vascular development, and the isolation of cloche is particularly difficult due to its telomeric location. Reischauer et al. systematically genome-edited each candidate genes in the cloche-containing region and successfully isolated the cloche gene, which greatly facilitated the following functional investigation of the gene in haemato-vascular specification pathways (Reischauer et al. 2016). These are typical case showing that genomic functional studies could be easily conducted with the combination of the CRISPR/Cas9 system and zebrafish.

In addition, multiplex biallelic genome editing can also be achieved simultaneously in CRISPR-edited zebrafish model (Jao et al. 2013). This can facilitate the recapitulation and observation of multiple phenotypes caused by multiple genes in the same clutch of fish, which is particularly important when a set of disease-associated genes are concurrently revealed by NGS studies or gene–gene interactions are under investigation. Moreover, multiplex conditional mutagenesis is particularly important to investigate the function of genes in a tissue-specific manner. Instead of injecting synthesized Cas9 and sgRNAs, these two elements are incorporated into the fish genome downstream of tissue-specific promoters. Similarly, with the Cas9 and sgRNA generated in the same cells, DSBs are induced and genome editing can be accomplished (Yin et al. 2015). Due to the ease of large-scale screening in zebrafish, CRISPR/Cas9-edited zebrafish model can expand the capacity of genome editing to study human genetic disease in a network-based approach (Barabasi et al. 2011; Jao et al. 2013; Shah et al. 2015; Varshney et al. 2015).

## Future development and promises

The combination of CRISPR-Cas9 system and zebrafish holds great promise for studying human genetic diseases. By various GWAS and exome sequencing studies, candidate disease genes are being identified continuously, of which the function needs to be validated in an easy and fast approach. Zebrafish is an ideal biological system in this case particularly considering its similarity to human biology and unusual speed to perform effective functional studies and drug discovery (Fig. 4). Based on the gene-related hypothesis of the disease, sgRNAs could be constructed and injected into fish stage embryos. In contrast, with limited information of candidate genes or mutations, sgRNA library is also easily constructed in the 96-well format with one target-specific sequence and the other a generic oligonucleotide that contains the remaining nucleotides required in a sgRNA (Varshney et al. 2015). Various mutant fish are conveniently identified and phenotypes of a certain disease model are characterized and categorized in details, referring to the clinical symptoms and signs observed in patients (Fig. 3). Finally, chemical screening is ready to be performed in a phenotype-based approach, as described in previous studies (Kokel et al. 2010; Kawahara et al. 2011; Baraban et al. 2013).

Better mutagenesis is expected with the optimization of the zebrafish CRISPR technology. Engineered Cas9 slightly different from that used in other systems has shown better expression and nuclear localization, thus contributing to higher efficiency (Jao et al. 2013). Similarly, modifications in the sgRNA sequence are also shown to improve mutagenesis rates (Hwang et al. 2013; Jao et al. 2013). However, genome-wide unbiased evaluation of the off-target has not been performed for CRISPR/Cas9 in the zebrafish, and unidentified off-target modifications in the fish genome may cause false-positive annotation of the gene functions. Despite the promising results, demonstration of the same high levels of mutagenesis accuracy and efficiency across a wider range of genes and phenotypes is needed. Moreover, various genetic perturbations, including knock-out, knock-down and over-expression, are complementary methods to obtain a comprehensive understanding of the causal links between genes and phenotypes. Combined with other traditional tools in zebrafish such as MO, possibilities of using CRIPSR/Cas9 systems still need to be extended with improvement in the technology and study designs.

## Conclusion

The wide use of next-generation sequencing has caused explosive identification of potential disease-causing variants. The proper design to understand the function of genetic elements in biological systems is necessary. Recently, zebrafish has become the trending animal model for investigating human genetic variants and diseases, supported by its genetic similarity to human and outstanding manipulability. Traditionally, zebrafish is the preferred model for studying vertebrate development, and its abundant research background comes with a number of technologies including gene-editing tools and integrated phenotyping methods. The recent three years has witnessed the sea change CRISPR/Cas9 has created in our ability to perform targeted gene perturbations in zebrafish, with high levels of on-target efficiency and relatively low off-target modifications. In addition, the CRIPSR/Cas9 derived toolbox is still under active development, and more comprehensive designs can be available with the introduction of conditional gene modification, multiplex biallelic genome editing and dCas9-medicated transcriptional regulations. High-throughput screens for phenotypic effects are facilitated by the combination of CRISPR/Cas9 systems and zebrafish, thus benefiting therapy development.

In conclusion, CRISPR/Cas9-editing in zebrafish is a reliable and promising method for genetic diseases modeling and medical genetic research.
